# Improved symptoms, exercise capacity, and homogeneity of cardiac deformation through conduction system pacing in a patient with symptomatic left bundle branch block

**DOI:** 10.1016/j.hrcr.2022.10.013

**Published:** 2022-10-19

**Authors:** Daniel Hofer, Shehab Anwer, Felix C. Tanner, Christoph Auf der Maur, Jan Steffel, Sergio Richter, Alexander Breitenstein

**Affiliations:** ∗ Department of Cardiology, University Hospital Zurich, Zurich, Switzerland; † Kardiologie Auf der Maur, Lucerne, Switzerland; ‡ Division of Electrophysiology, Department of Cardiology, Heart Center Dresden, Technische Universität Dresden, Dresden, Germany

**Keywords:** Left bundle branch block, Symptomatic, Painful, Conduction system pacing, His pacing

**Key Teaching Points**

• The painful left bundle branch block (LBBB) syndrome is a potentially underestimated and underreported disease with potentially debilitating symptoms.
• In patients with anginal symptoms, LBBB, and absence of other causative diseases, painful LBBB should be considered.
• His bundle pacing may ameliorate symptoms, increase exercise capacity, and improve homogeneity of cardiac deformation.

## Introduction

Painful left bundle branch syndrome is a clinical entity consisting of exertional angina and rate-dependent left bundle branch block (LBBB), affecting patients of all age and sex.^1^ Because of potentially coexisting other cardiac diseases (ie, cardiomyopathy, coronary artery disease) that may mimic both LBBB and symptoms, the true prevalence is unknown, but fewer than 60 cases have been reported so far.^1^ Diagnostic criteria do not officially exist, but simultaneous onset of LBBB and angina during exercise test support the diagnosis. Pacemaker implantation has been reported to alleviate symptoms; however, high percentages of right ventricular pacing may induce cardiomyopathy, whereas implantation of a cardiac resynchronization therapy device carries an additional short- and long-term complication risk. Conduction system pacing may present a safe and effective alternative pacing method,^2^ but the effect of this type of pacing in patients with painful LBBB syndrome has not yet been studied. We report a case of painful LBBB with symptom amelioration, increased exercise capacity, and improved homogeneity of cardiac deformation after implantation of a His bundle pacemaker (HBP).

## Case report

**Figure 1:**
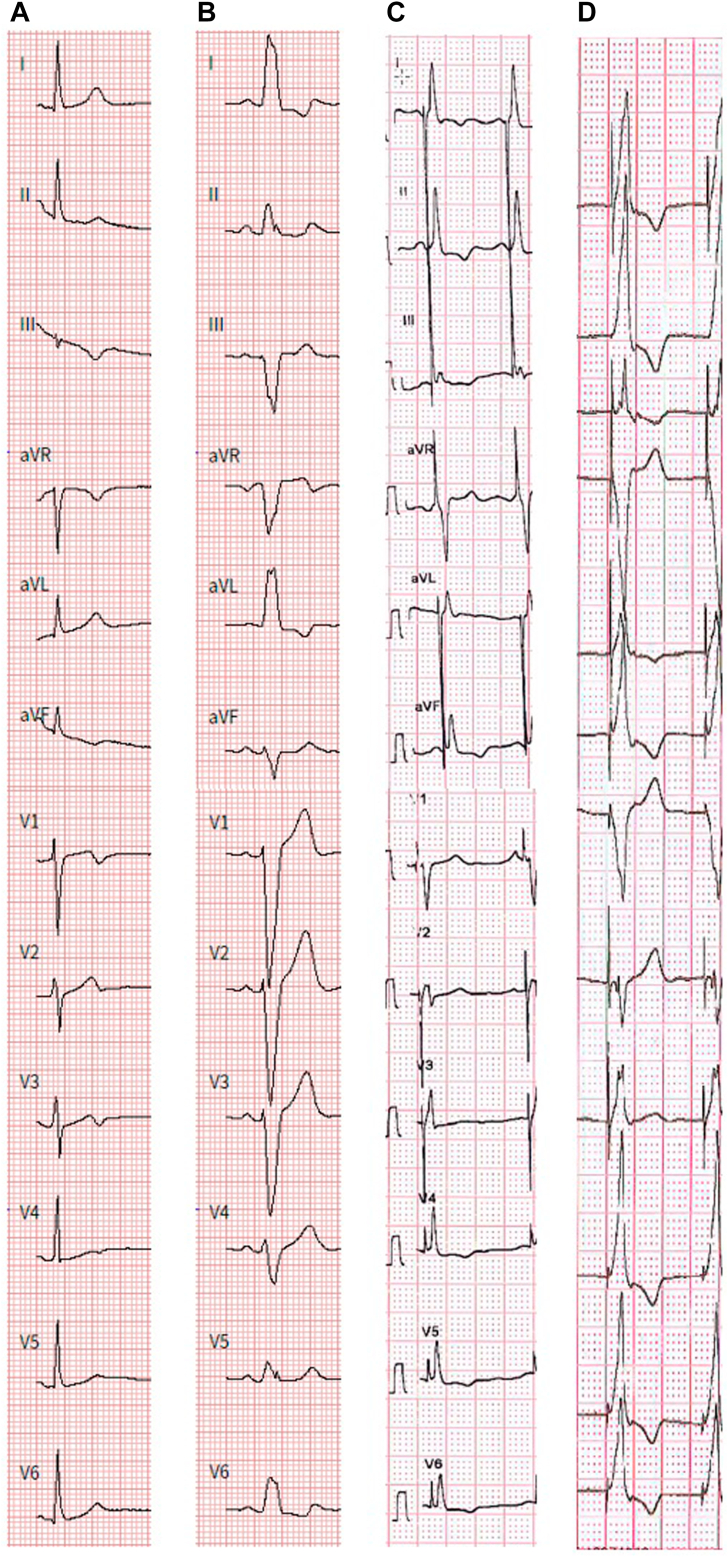
Electrocardiograms during sinus rhythm and His lead pacing. **A:** Sinus rhythm without left bundle branch block (LBBB), QRS 84 ms. **B:** Sinus rhythm with LBBB starting at a heart rate of 75 beats/min, QRS 153 ms. **C:** His lead pacing resulting in selective His capture with correction of bundle branch block at a unipolar threshold of 2 V / 1 ms, QRS 92 ms. **D:** His lead pacing resulting in selective His capture without correction of bundle branch block at a unipolar threshold of 0.9 V / 1 ms, QRS 149 ms.

**Figure 2:**
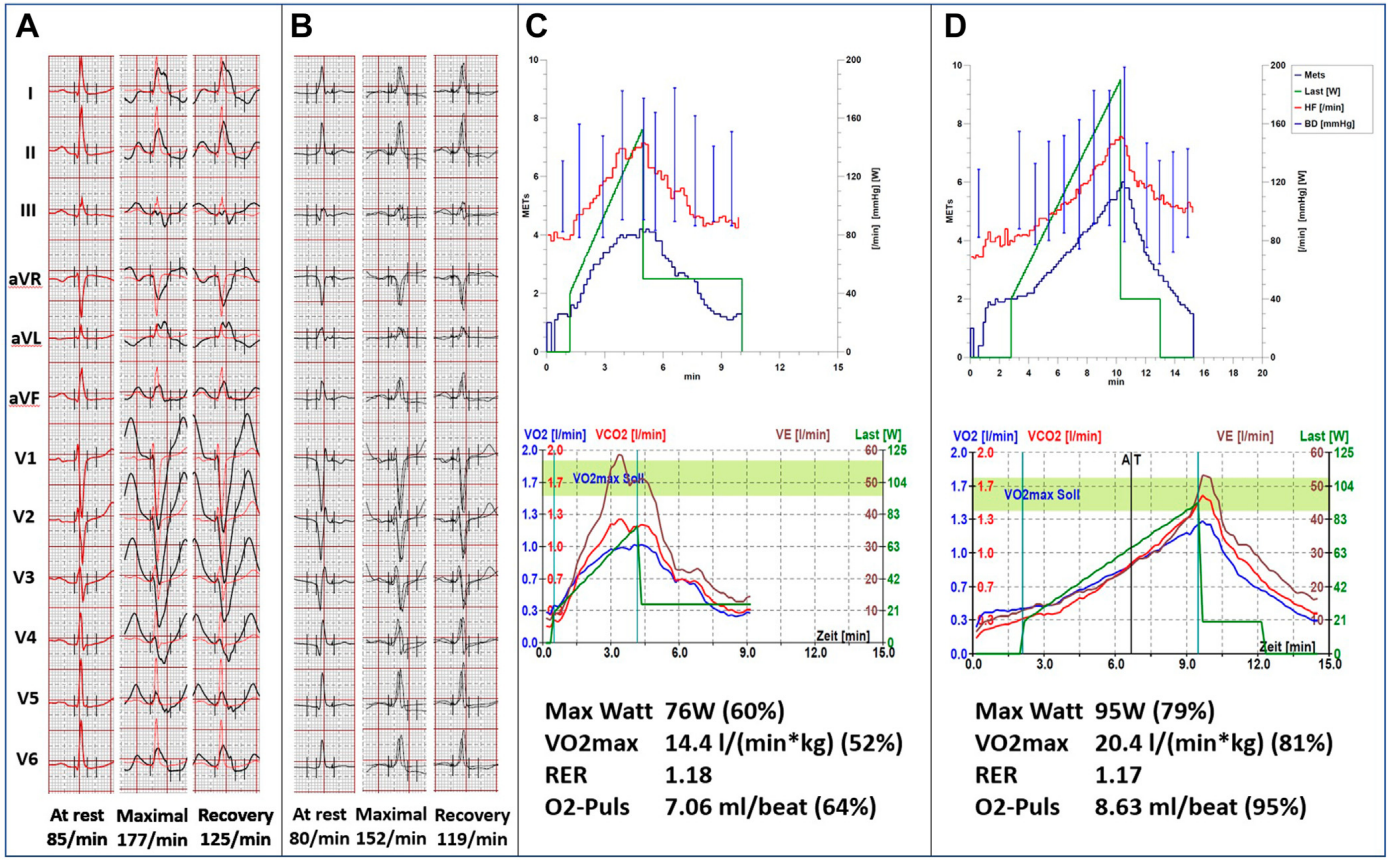
Exercise test before and after His bundle pacemaker (HBP) implantation. **A:** Electrocardiograms (ECGs) during exercise test before HBP implantation. **B:** ECGs during exercise test after HBP implantation. **C:** Exercise test before HBP implantation. **D:** Exercise test after HBP implantation. % refers to age- and sex-adjusted mean.

A 45-year-old female patient was referred to our institution for evaluation of progressive exercise intolerance associated with crushing thoracic pain and breathlessness. Symptoms started 9 years prior with occurrence during strong exercise but were progressive for the last years to the extent of restricting everyday exercise capabilities such as grocery shopping. Previous medical work-up revealed no pulmonary cause, while cardiac evaluation documented an intermittent LBBB with normal echocardiographic evaluation. Previous empirical therapy with a calcium channel blocker for suspected vasospastic angina or microcirculatory ischemia and with bronchodilator inhalation for suspected bronchial asthma was unsuccessful. Also, cardiac rehabilitation with daily exercise sessions had not yielded beneficial results. During normal sinus rhythm (heart rate below 75 beats per minute [bpm]), no bundle branch block or repolarization abnormalities were noted. Holter monitoring, however, revealed rate-dependent LBBB coinciding with the patient’s symptoms starting at a heart rate of 75 bpm (Figure 1). Exercise stress testing demonstrated reduced exercise capacity (76 W = 60% of age- and sex-adjusted mean, VO2 max 14.4 mL/min/kg = 52% of age- and sex-adjusted mean) with a decreased maximal O2 pulse of 7 mL/beat (= 64% of age- and sex-adjusted mean), with the LBBB being present throughout the examination (Figure 2). Cardiac magnetic resonance imaging was unremarkable without evidence of ischemia or fibrosis. However, both magnetic resonance imaging and echocardiography demonstrated typical dyssynchronous motion of the interventricular septum during LBBB. Electrophysiologic study revealed normal intracardiac timing intervals (His ventricle time 48 ms) both with and without LBBB, with the latter manifesting below an atrial pacing cycle length of 830 ms. Pacing at the area of the His bundle revealed nonselective His capture (stimulation of the His bundle as well as the surrounding myocardium) with a narrow QRS (78 ms) without evidence of LBBB. Following the diagnosis of symptomatic left bundle branch syndrome and extensive discussion about the risks and benefits of the available treatment options, the patient underwent successful dual-chamber HBP implantation using a SelectSecure 3830 electrode (Medtronic, Minneapolis, MN) through a nondeflectable C315 sheath (Medtronic). Continuous selective His capture with correction of LBBB was documented with a unipolar threshold of 0.9 mV / 1.0 ms. Rate-dependent atrioventricular delay shortening was programmed, resulting in His stimulation only at atrial rates above 75 bpm. After 3 months, the patient reported significant reduction of symptoms and nearly no further limitations during daily activities. Device interrogation revealed 24% of ventricular pacing over time with a unipolar threshold of 2 V / 1 ms for selective His capture with LBBB correction and a unipolar threshold of 0.9 V / 1 ms for selective His capture without LBBB correction (Figure 1).

**Figure 3:**
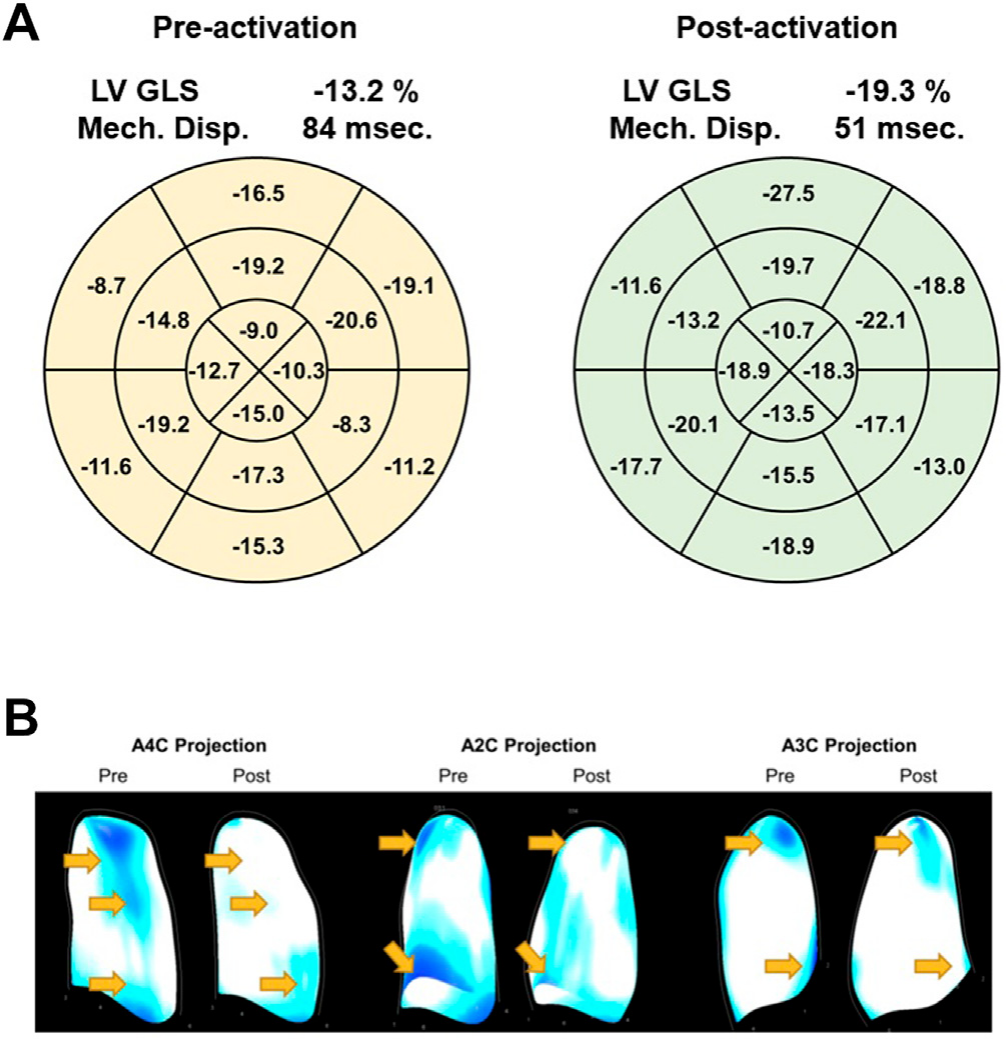
**A:** Left ventricular longitudinal strain without (left panel, pacemaker programmed to AAI 100/min) and with (right panel, pacemaker programmed to DDD 100/min with left bundle branch block [LBBB] correction) His pacing demonstrating improved global and segmental strain and mechanical dispersion. LV GLS = left ventricular global longitudinal strain; Mech. Disp. = mechanical dispersion. **B:** Three-dimensional simulation of left ventricular strain map during systole showing improved regional strain as observed from the apical view. A2C = 2-chamber view; A3C = 3-chamber view; A4C = 4-chamber view; Post = with His bundle pacing, pacemaker programmed to DDD 100/min with LBBB correction; Pre = without His bundle pacing, pacemaker programmed to AAI 100/min.

Repeat exercise test demonstrated improved exercise capacity (95 W = 79% vs previously 76 W = 60% of age- and sex-adjusted average) and improved peak oxygen uptake (VO2 max 20.4 mL/min/kg vs previously 14.4 mL/min/kg). During echocardiographic evaluation offline strain analysis with 3-dimensional rendering of left ventricular deformation was performed using TomTec ImageArena Cardiac Performance Analysis module (v.4.6). Global and segmental strain values were described based on a 16-segment left ventricular model from tracking the left ventricular endocardial border in the apical 2-, 3-, and 4-chamber views according to current recommendations.^3^ Echocardiographic analysis during LBBB vs His bundle-pacing demonstrated improvement in segmental and global longitudinal strain (-13.2% vs -19.3%, respectively; Figure 3A), as well as improved mechanical dispersion (84 ms vs 51 ms, respectively; Figure 3), indicating increased ventricular deformation and synchronicity of contraction. Similarly, 3D simulation of left ventricular strain map during systole visualized improved ventricular deformation (Figure 3B, Supplemental Videos 1,2,3,4). After 12 months, the patient reported no limitations anymore during daily activities or cardiovascular exercise. She had reduced her body mass index from 27 to 24 kg/m^2^ within 1 year owing to increased capacity during cardiovascular exercise. Device interrogation demonstrated a stable pacing percentage of around 25% and a stable unipolar threshold of 2 V / 1 ms for selective His capture with LBBB correction.

## Discussion

The “Painful LBBB syndrome” consists of anginal symptoms and rate-dependent LBBB during exertion, first published more than 70 years ago.^4^ In the largest published series of 50 patients, there seems to be no sex or age preponderance, a characteristic electrocardiographic LBBB pattern with a low S/T wave ratio, and—most importantly—a good long-term prognosis.^1^ Substantial interpatient variability is observed, with reported symptoms from “heart throbbing” to “debilitating pain limiting everyday physical activity.”^1^ Our patient reported a progressive nature of her symptoms over several years, experiencing symptoms even during simple daily activities at the time of referral to our clinic.^5^^,^^6^ LBBB and associated symptoms appear to be easily reproducible in such patients by physical activity, atrial pacing, or pharmacological challenge (ie, application of isoproterenol, atropine), and symptoms usually vanish with resolution of LBBB below a certain heart rate. It is fundamental to exclude a relevant underlying structural or functional heart disease, as other cardiac conditions may present with similar symptoms and electrocardiographic patterns. Mechanistically, previous reports have excluded ischemia as the causative mechanism of painful LBBB,^1^ while current pathophysiologic theories favor dyssynchronous cardiac ventricular contraction and increased interoceptive sensitivity.^1^^,^^7^

While no specific treatment protocols for symptomatic LBBB exist, therapeutic options include a physical exercise regimen to increase the rate threshold of LBBB onset, beta blockers to limit heart rate, or pacemaker implantation.^1^^,^^8^ In our patient, physical training had no effect and beta-blocker therapy was not tolerated; therefore we jointly decided for a pacemaker implantation. Even though right ventricular and biventricular pacing have both been reported to be successful in alleviating symptoms in patients with painful LBBB,^9^ chronic right ventricular pacing is known to be a risk factor for pacing-induced cardiomyopathy,^10^ while CRT implantation carries an increased risk of short- and long-term complications. His bundle pacing presents a valid alternative to restore cardiac electrical synchronicity through physiological stimulation.^11^ Because of the longitudinal electrical dissociation within the His bundle, LBBB with proximal or intrahisian origin may be overcome with direct His bundle pacing.^12^^,^^13^ To estimate the level of electrical block in patients with painful LBBB syndrome, an electrophysiological study is warranted, where nonselective His capture with successful correction of bundle branch block was documented in our patient. Hence, His bundle pacing with correction of LBBB was deemed feasible and an HBP was successfully implanted. Similarly, His bundle pacing has been reported as a successful therapy in painful LBBB syndrome in at least 4 other case reports.^6^^,^^14^^,^^15^

However, the effect of His bundle pacing on objective parameters including exercise capacity and echocardiographic parameters of ventricular electrical synchrony in this type of patients has, to the best of our knowledge, not yet been reported. A higher exercise capacity (110 W = 89% vs previously 76 W = 60% of age- and sex-adjusted mean) and peak oxygen uptake (VO2 max 18.4 mL/min/kg vs previously 14.4 mL/min/kg) after HBP implantation was documented in our patient, corresponding well to her significantly improved symptoms. Echocardiographic evaluation demonstrated improved global left ventricular strain, improved electrical dispersion, and improved homogeneity of cardiac contraction (Figure 3, Supplemental Videos 1,2,3,4), underscoring the hypothesis of dyssynchronous ventricular contraction being corrected by HBP in this population.

## Conclusion

Painful LBBB syndrome is a potentially underestimated and underreported disease with potentially debilitating symptoms. In patients with anginal symptoms, LBBB, and absence of other causative diseases, painful LBBB should be considered. His bundle pacing may ameliorate symptoms, increase exercise capacity, and improve homogeneity of cardiac deformation.

## Appendix Supplementary Data

Supplemental Video 1 3D simulation of left ventricular strain map during systole observed from the four chamber view without His-bundle-pacing (pacemaker programmed to AAI 100/min).

Supplemental Video 2 3D simulation of left ventricular strain map during systole observed from the four chamber view with His-bundle-pacing (pacemaker programmed to DDD 100/min with correction of left bundle branch block) demonstrating improved regional strain.

Supplemental Video 3 3D simulation of left ventricular strain map during systole observed from the two chamber view without His-bundle-pacing (pacemaker programmed to AAI 100/min).

Supplemental Video 4 3D simulation of left ventricular strain map during systole observed from the two chamber view with His-bundle-pacing (pacemaker programmed to DDD 100/min with correction of left bundle branch block) demonstrating improved regional strain.

## References

[1] Shvilkin A., Ellis E.R., Gervino E.V., Litvak A.D., Buxton A.E., Josephson M.E. (2016). Painful left bundle branch block syndrome: clinical and electrocardiographic features and further directions for evaluation and treatment. Heart Rhythm.

[2] Abdelrahman M., Subzposh F.A., Beer D. (2018). Clinical outcomes of His bundle pacing compared to right ventricular pacing. J Am Coll Cardiol.

[3] Voigt J.U., Pedrizzetti G., Lysyansky P. (2015). Definitions for a common standard for 2D speckle tracking echocardiography: consensus document of the EACVI/ASE/Industry Task Force to standardize deformation imaging. Eur Heart J Cardiovasc Imaging.

[4] Eichert H. (1946). Transient bundle branch block associated with tachycardia. Am Heart J.

[5] Hertzeanu H., Aron L., Shiner R.J., Kellermann J. (1992). Exercise dependent complete left bundle branch block. Eur Heart J.

[6] Suryanarayana P.G., Frankel D.S., Marchlinski F.E., Schaller R.D. (2018). Painful left bundle branch block [corrected] syndrome treated successfully with permanent His bundle pacing. HeartRhythm Case Rep.

[7] Cannon R.O., Quyyumi A.A., Schenke W.H. (1990). Abnormal cardiac sensitivity in patients with chest pain and normal coronary arteries. J Am Coll Cardiol.

[8] Heinsimer J.A., Skelton T.N., Califf R.M. (1986). Rate-related left bundle branch block with chest pain and normal coronary arteriograms treated by exercise training. Am J Med Sci.

[9] Sroubek J., Tugal D., Zimetbaum P.J., Shvilkin A., Buxton A.E. (2019). Treatment of painful left bundle branch block syndrome with cardiac resynchronization therapy or right-ventricular pacing. HeartRhythm Case Rep.

[10] Merchant F.M., Mittal S. (2020). Pacing induced cardiomyopathy. J Cardiovasc Electrophysiol.

[11] Lustgarten D.L., Calame S., Crespo E.M., Calame J., Lobel R., Spector P.S. (2010). Electrical resynchronization induced by direct His-bundle pacing. Heart Rhythm.

[12] Tung R., Upadhyay G.A. (2020). Defining left bundle branch block patterns in cardiac resynchronisation therapy: a return to His bundle recordings. Arrhythm Electrophysiol Rev.

[13] Upadhyay G.A., Cherian T., Shatz D.Y. (2019). Intracardiac delineation of septal conduction in left bundle-branch block patterns. Circulation.

[14] Viles-Gonzalez J.F., Mahata I., Anter E., d'Avila A. (2018). Painful left bundle branch block syndrome treated with his bundle pacing. J Electrocardiol.

[15] Oladunjoye O.O., Oladunjoye A.O., Oladiran O., Callans D.J., Schaller R.D., Licata A. (2019). Persistent exertional chest pain in a marathon runner: exercise-induced, painful, left bundle branch block syndrome treated with His-bundle pacing. Mayo Clin Proc Innov Qual Outcomes.

